# Serum Serine as a Metabolic Marker of Optic Neuropathy in Patients with Type 2 Diabetes Mellitus After Vitreoretinal Surgeries

**DOI:** 10.3390/metabo16090622

**Published:** 2026-08-28

**Authors:** Oleksii Ivashyn, Volodymyr Gryshchuk, Vita Ivashyna, Maryna Miasnykova, Heorhii Yurlov, Svitlana Stepanenko, Dmytro Zhaboiedov

**Affiliations:** 1 Department of Ophthalmology, Bogomolets National Medical University, 01601 Kyiv, Ukraine; dmitriyg.med@gmail.com; 2 Department of Adult and Pediatric Ophthalmology, Shupyk National Healthcare University of Ukraine, 04112 Kyiv, Ukraine; 3 Department of Protein Structure and Function, Palladin Institute of Biochemistry of NAS of Ukraine, 01054 Kyiv, Ukraine; 4 Chromatography Centre, Palladin Institute of Biochemistry of NAS of Ukraine, 01054 Kyiv, Ukraine; myasnikova@biochem.kiev.ua (M.M.); yurlov@biochem.kiev.ua (H.Y.); sst@biochem.kiev.ua (S.S.); 5 Department of Pathology of Premature and Newborns, Kyiv Regional Children’s Hospital, 08150 Boyarka, Ukraine; vitaivashina86@gmail.com

**Keywords:** type 2 diabetes, serine, optic neuropathy, optical coherence tomography, automated perimetry, retinal nerve fiber layer, amino acid analysis, vitrectomy

## Abstract

**Background**: Optical neuropathy is a severe complication in patients with type 2 diabetes mellitus (T2DM) undergoing vitreoretinal surgeries. Early diagnostic indicators are critical to preventing irreversible vision loss. This study aimed to evaluate the alterations in blood serum amino acid profiles, with a specific focus on serine levels, and their potential clinical association with the severity of neuroretinal impairment. **Methods**: The clinical cohort included 28 patients aged 32 to 77 years with T2DM following vitreoretinal interventions. Visual field functional status was assessed using Humphrey automated perimetry, with mean deviation (MD), pattern standard deviation (PSD), and visual field index (VFI). Structural changes were quantified using optical coherence tomography (OCT) to measure peripapillary retinal nerve fiber layer (RNFL) thickness. Serum amino acid profiles were determined using an ion-exchange chromatography amino acid analyzer, alongside a reference group (*n* = 12). **Results**: All patients presented with moderate-to-severe visual field impairment. A profound decline in serum serine levels was observed in diabetic patients compared to the reference group (*p* < 0.001). The reduction in serine content closely mirrored both the degree of retinal sensitivity loss (MD and PSD values) and the number of thinned peripapillary RNFL sectors. Notably, the compensatory synthesis of serine via glycine cleavage appeared insufficient, as evidenced by a concomitant statistical decrease in glycine content (*p* < 0.05). No significant differences in serine levels were found between the subgroups when stratified strictly by age or isolated severity stages. **Conclusions**: It was demonstrated that the decline in serum serine levels in patients with T2DM following vitreoretinal interventions is significantly associated with the severity of optic neuropathy as assessed by optical coherence tomography (OCT). Consequently, serum serine may serve as a valuable dual biomarker, acting as a prognostic indicator for retinopathy progression and a predictive marker for early-stage neuropathy development.

## 1. Introduction

Recent epidemiological data indicate that the global prevalence of diabetes mellitus is projected to reach 643 million cases by 2030. Consequently, diabetes mellitus represents one of the major and fastest-growing global health challenges [1]. Hence, the prevention and management of diabetic complications pose a substantial burden on healthcare systems. The most prevalent complication is diabetic neuropathy (DN), a neurodegenerative disorder affecting the peripheral nervous system. The disease predominantly affects sensory neurons and is broadly classified into diffuse and focal forms [1,2,3]. The most common phenotype of DN is distal symmetric polyneuropathy (DSPN), a subtype of diffuse diabetic peripheral neuropathy (DPN), which accounts for approximately 75% of all diabetic neuropathy cases [4]. Diabetic autonomic neuropathy (DAN) affects the autonomic fibers and can induce severe dysfunction across multiple organ systems [3]. Additionally, DAN can present in atypical forms, including mononeuropathy and radiculopathy/polyradiculopathy [1,2,3]. Insulin resistance, hyperglycemia, and dyslipidemia act on neurons, glial cells, and vascular endothelial cells via distinct signaling pathways. This process triggers DNA damage, mitochondrial impairment, and endoplasmic reticulum stress, ultimately leading to apoptosis and the loss of neurotrophic support [5,6]. Although the onset and progression of diabetic comorbidities are primarily attributed to these factors, abnormal metabolism of non-essential amino acids (NEAAs) also significantly contributes to the pathogenesis of diabetes. Serine and glycine are closely linked NEAAs whose levels are consistently diminished in patients with metabolic syndrome [7,8]; however, the mechanistic drivers and consequences of this metabolic dysregulation remain poorly understood. Low systemic levels of serine and glycine are also hallmarks of macular and peripheral nervous system disorders, correlating with impaired visual acuity and peripheral neuropathy [9,10,11]. Consequently, patients with type 2 diabetes mellitus should undergo an initial dilated and comprehensive eye examination by an ophthalmologist or optometrist at the time of diagnosis [12]. Accordingly, the present study aims to analyze the amino acid composition of blood serum in patients of various ages with type 2 diabetes following vitreoretinal interventions.

## 2. Materials and Methods

### 2.1. Materials

Amino Acid Standard, analytical standard, ninhydrin, dimethyl sulfoxide, sulfosalicylic acid, lithium hydroxide, lithium chloride, citric acid anhydrous, lithium citrate, concentrated hydrochloric acid (Thermo Fisher Scientific Inc., Waltham, MA, USA), and ion-exchange resins for amino acid analysis were obtained from OSTION POLY 8 (INGOS Ltd., Praha, Czech Republic).

### 2.2. Patients

A total of 28 patients with type II diabetes mellitus who underwent vitreoretinal surgery, aged 32 to 77 years ((M ± SD) = 58.89 ± 11.76 years), were included in the study.

Inclusion Criteria:

- Obtained permission to access and process the medical histories of operated patients;
- Signed informed consent from the patient to participate in the study;
- Patients with type 2 diabetes mellitus who underwent vitreoretinal surgery;
- Patients with rhegmatogenous retinal detachment who underwent vitreoretinal surgery;
- Age between 30 and 90 years.

Exclusion Criteria (at baseline):

- Patient’s refusal to participate in the study;
- Age outside the specified range;
- Concomitant glaucoma;
- A history of acute ischemic optic neuropathy or acute optic nerve blood supply disorders;
- A history of central retinal vein or artery occlusion;
- Optic nerve atrophy of any etiology.

Withdrawal Criteria (during the study):

- Non-compliance with the study protocol;
- Postoperative detection of concomitant ocular pathology, including acute optic nerve ischemia, glaucoma, central retinal vein or artery occlusion, or toxic optic neuropathy;
- Patient’s withdrawal of consent at any stage of the study;
- Pregnancy.
- In the event of patient withdrawal, additional subjects were recruited to meet the inclusion criteria.
- The cohort primarily comprised individuals from the central and western regions of Ukraine.

All participants underwent a comprehensive ophthalmological examination at baseline and during follow-up. Best-corrected visual acuity (BCVA) was assessed using standardized charts (Decimal Snellen fractions AP-1M, Zapovit, Kyiv, Ukraine) following objective refractometry (Topcon KR-800, Topcon Corporation, Tokyo, Japan) to ensure optimal correction. Intraocular pressure (IOP) was measured using a non-contact tonometer (Topcon CT-80, Topcon Corporation, Tokyo, Japan). Functional assessment of the optic nerve was performed using automated static perimetry (Humphrey Field Analyzer 3, Carl Zeiss Meditec, Jena, Germany), with analysis of mean deviation (MD) and pattern standard deviation (PSD), where higher values indicated more severe visual field impairment. Structural evaluation was carried out using optical coherence tomography and OCT angiography (Optopol REVO, Optopol Technology, Zawiercie, Poland), with retinal nerve fiber layer (RNFL) thickness quantified across 12 sectors and interpreted using device-provided normative color coding. Anterior and posterior segment examinations were performed using slit-lamp biomicroscopy (Topcon SL-1E, Topcon Corporation, Tokyo, Japan) and ophthalmoscopy (Welch Allyn PanOptic, Skaneateles Falls, NY, USA), with optic disc appearance used as a qualitative indicator of optic nerve atrophy.

All patients enrolled in the study were hospitalized prior to the intervention and prescribed a carbohydrate-controlled diet with restricted intake of easily digestible carbohydrates.

All work was conducted in accordance with the Declaration of Helsinki. Studies were conducted according to the Ethical Committee Approval N 2/4 form 10.01.2025 (Shupyk National Healthcare University of Ukraine, 04112, Kyiv, Ukraine). Informed consent was obtained from all subjects involved in the study.

### 2.3. Methods

#### 2.3.1. Humphrey Field Analysis

Visual fields were assessed using the Humphrey Field Analyzer 3 (Carl Zeiss Meditec, Dublin, CA, USA) with the 30-2 threshold program. The perimetric indices were evaluated [13,14]:

- Mean deviation (MD): The average difference between the patient’s visual field and the age-matched normative database, expressed in decibels (dB). Values ranging from −2 dB to +2 dB were considered normal, whereas negative values indicated a reduction in global retinal sensitivity.
- Pattern standard deviation (PSD): The degree of localized departure of the patient’s retinal sensitivity from the expected normal visual field hill of vision, measured in decibels (dB). A value of 2 dB was defined as normal, with higher values indicating localized visual field defects (scotomas).
- Visual field index (VFI): A global index expressed as a percentage (%), where 100% represents a completely normal visual field and 0% represents a total loss of visual field sensitivity [15].

#### 2.3.2. Optical Coherence Tomography (OCT)

Peripapillary retinal nerve fiber layer (RNFL) thickness was measured using the Optopol REVO optical coherence tomography system (Optopol Technology, Zawiercie, Poland). To account for the physiological age-related decline in RNFL thickness (approximately 0.52 µm/year), the following normative reference values for healthy adults were utilized:

- 20–39 years: 105.2 ± 7.1 µm;
- 40–59 years: 101.5 ± 5.8 µm;
- 60 years: 94.8 ± 6.6 µm (with physiological variations extending to 96.2 µm in the 80–89 age subgroup) [16].

The evaluated RNFL parameters included:

- Average RNFL thickness;
- Inter-eye symmetry (a comparison of RNFL thickness between the right and left eyes);
- Quadrants and sectors (the peripapillary region was segmented into Superior, Inferior, Nasal, and Temporal quadrants) [17,18].

Diagnostic classification was based on the device’s built-in normative database and color-coded scale:

- Green (within normal limits)—thickness values falling within the 95% percentile of the normal population;
- Yellow (borderline)—values within the 1% to 5% percentile, indicating a suspected pathology;
- Red (outside normal limits): values below the 1% percentile, representing pronounced thinning with a high probability of glaucoma or optic neuropathy.

#### 2.3.3. Blood Plasma and Serum Preparation

Platelet-poor blood plasma was prepared from citrated blood by centrifugation at 1200 *g* for 30 min. Sodium Citrate (3.8%) was added immediately after collection to whole blood at a 1:9 ratio as an anticoagulant [19].

Blood plasma serum was prepared from plasma using the thrombin-like enzyme ancistron [20]. This allowed fibrinogen to be removed without affecting other proteins of the blood coagulation system. Serum samples were centrifuged at 1200 *g* for 30 min.

#### 2.3.4. Hydrolysis of Samples

A weighed sample containing 50 ± 0.5 mg of dry protein, or an equivalent aliquot of an aqueous protein solution, was placed at the bottom of a heat-resistant glass tube. Five milliliters of concentrated hydrochloric acid was added to the protein sample, and the mixture was boiled for 30 min. The tube was then sealed with a ground-glass stopper and incubated in a thermostat at 106 °C for 24 h. Upon completion of hydrolysis, the tube was allowed to cool to room temperature before opening. The contents were quantitatively transferred to a glass weighing bottle and evaporated to dryness on a water bath. To remove residual acid, 4 mL of deionized water was added to the weighing bottle, and the drying procedure was repeated three times. The resulting residue was dissolved in 0.3 N lithium citrate buffer (pH 3.2) and loaded onto an OSTION POLY 8 ion-exchange column (INGOS Ltd., Praha, Czech Republic) of a T-339 Mikrotekno amino acid analyzer (INGOS Ltd., Praha, Czech Republic).

#### 2.3.5. Amino Acid Analysis

The test sample is diluted in a lithium citrate buffer (pH 2.2) and loaded onto the OSTION POLY 8 ion-exchange column (INGOS Ltd., Praha, Czech Republic). Amino acids are eluted from the ion-exchange column sequentially using lithium citrate buffer solutions with pH values of 2.75, 2.95, 3.2, 3.8, and 5.0. The ratio of the ninhydrin reagent to the eluent is 1:2, and the column oven temperature is maintained step-wise at 38.5 °C and 65 °C. For the quantitative determination of amino acids, a reference standard mixture with a known concentration of each amino acid is injected into the amino acid analyzer (T-339 Mikrotekno, INGOS Ltd., Praha, Czech Republic). The peak area for each amino acid is determined from the resulting chromatogram. The amount of each amino acid in µmol in the sample is calculated using the following Formula (1):

(1) $$X_{1}=\frac{S_{1}}{S_{0}}$$

where X_1_—the amount of each amino acid;

*S*_1_—the peak area of the analyte in the sample;

*S*_0_—the peak area of the standard amino acid corresponding to 1 µmol.

The weight is obtained by multiplying the micromoles of the amino acid by its respective molecular weight. The qualitative composition of the amino acid mixture is determined by comparing the retention times and chromatograms of the standard mixture with those of the test sample.

Reference group:

A total of 12 healthy control subjects aged 25 to 59 years ((M ± SD) = 41 ± 11 years), with no history of ophthalmological or neuropathic disorders or diabetes, were enrolled.

#### 2.3.6. Statistics

Prior to the comparative analysis, the normality of data distribution in all samples was assessed using the Shapiro–Wilk test. Given that the experimental groups varied in size and, in several instances, deviated from a normal distribution, both parametric and nonparametric statistical methods were applied. For normally distributed data, the results are expressed as the mean and standard deviation (M ± SD). For samples with a non-normal distribution, the data are presented as the median (Me) and interquartile range (Q1–Q3). Intergroup differences were evaluated using either Student’s *t*-test or one-way analysis of variance (ANOVA) followed by appropriate post hoc tests (in the case of normal distribution and homogeneity of variances) or the nonparametric Mann–Whitney U test (in the case of deviation from normality). All assays were performed in triplicate. Statistical significance was set at (*p* < 0.05).

## 3. Results

### 3.1. Patient Cohort Characterization

The study enrolled a cohort of 28 patients aged 32 to 77 years with a history of type 2 diabetes who had undergone vitreoretinal interventions. Visual field evaluation was performed to determine standard automated perimetry indices, including mean deviation (MD), pattern standard deviation (PSD)—which reflects the degree of localized reduction in retinal sensitivity relative to the normal reference pattern—and the visual field index (VFI). The presence of optic neuropathy was diagnosed using optical coherence tomography (OCT). Based on the mean values of the perimetric indices and the number of thinned peripapillary retinal nerve fiber layer (RNFL) segments, all patients presented with moderate-to-severe impairment of ocular functional status (Table 1).

### 3.2. Overall Amino Acid Analysis of Serum Samples

In the subsequent phase, the amino acid profiles of the blood serum were analyzed across the entire patient cohort. Amino acid levels were expressed as percentages relative to the total amino acid pool (Table 2).

As shown in Table 2, amino acid metabolism in the patient cohort was markedly altered; however, particular emphasis should be placed on the changes in serine levels, which may serve as an early biomarker of retinal neuropathy in patients with type 2 diabetes.

### 3.3. Serine Level Correlation with Clinical Parameters

**Figure 1:**
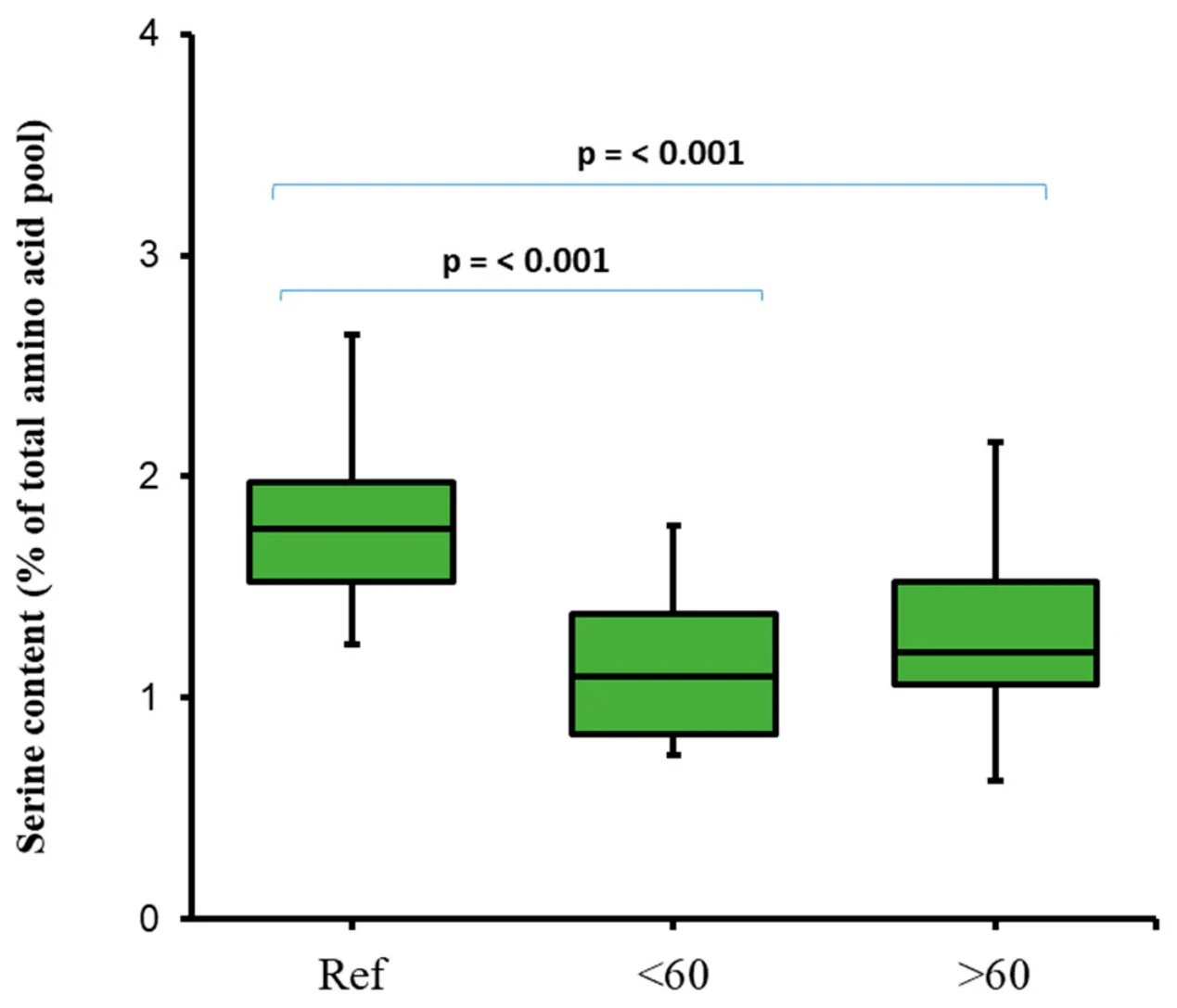
Serine levels in the blood serum of patients with type 2 diabetes following vitreoretinal interventions. Ref, reference group; <60, patients aged under 60 years; >60, patients aged over 60 years.

Previous studies have demonstrated that age plays a critical role in the development of sensory neuropathy in type 2 diabetes [21]. Therefore, the potential impact of patient age on serine levels was evaluated. Patients were stratified into two age groups: under 60 years and over 60 years. As illustrated in Figure 1, a distinct pattern emerged in the changes in serine levels across the experimental groups relative to the reference group; however, no statistically significant difference in serine levels was observed between the two age groups.

The subsequent objective of this study was to evaluate the dependence of blood serum serine levels on the severity of visual field impairment using Humphrey automated perimetry based on three standard perimetric indices [13,14].

**Figure 2:**
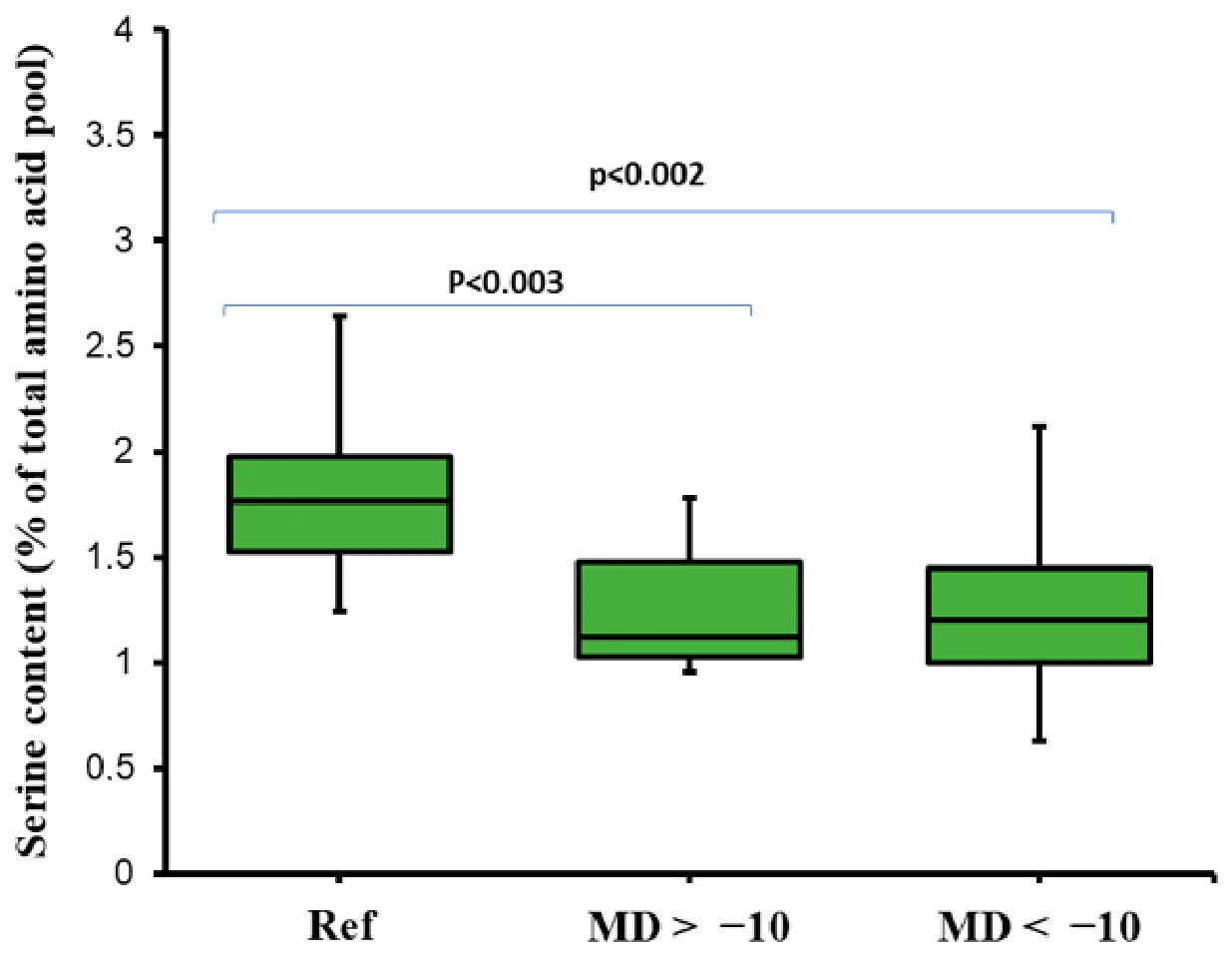
Serine levels in the blood serum of patients with type 2 diabetes following vitreoretinal interventions compared to the reference group, stratified by the severity of retinal sensitivity loss. Ref, reference group; MD > −10 dB, patients with moderate retinal sensitivity loss; MD < −10 dB, patients with severe retinal sensitivity loss.

The trends across all three perimetric indices were consistent, revealing a distinct association between the reduction in serine levels and the degree of retinal sensitivity loss compared to the reference group. However, consistent with the previous findings, no statistically significant differences in serine levels were observed between the subgroups stratified by the severity of visual field impairment (Figure 2).

**Figure 3:**
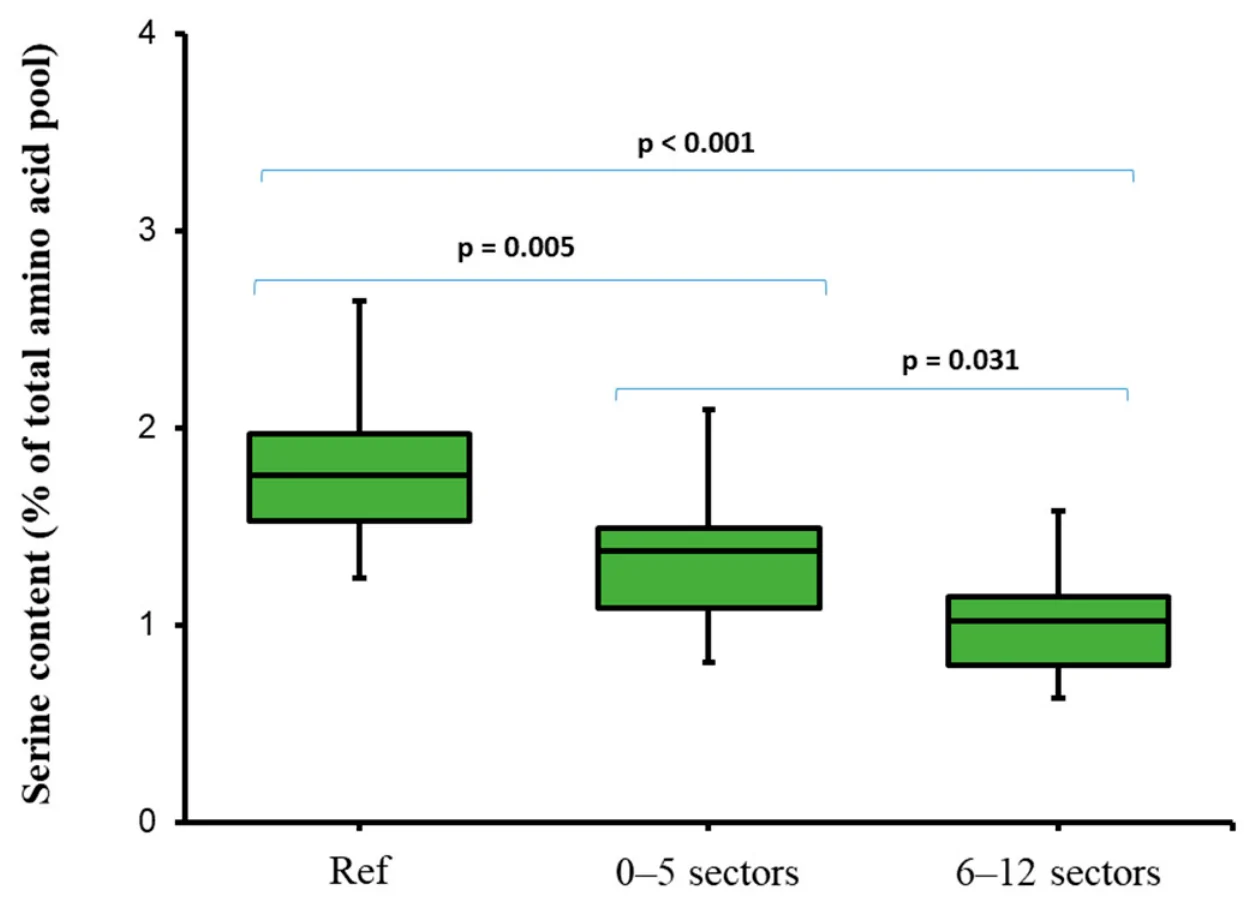
Serine levels in the blood serum of patients with type 2 diabetes following vitreoretinal interventions compared to the reference group, stratified by the severity of optic neuropathy based on optical coherence tomography (OCT) findings. Ref, reference group; 0–5 sectors, patients with moderate optic neuropathy; 6–12 sectors, patients with severe optic neuropathy.

Given that Humphrey automated perimetry exhibits lower sensitivity, particularly in patients who have undergone panretinal laser photocoagulation, the thickness of the peripapillary retinal nerve fiber layer (RNFL) was evaluated using optical coherence tomography (OCT) to accurately quantify the severity of neuropathy. As illustrated in Figure 3, a distinct dependence of serine levels was observed not only relative to the reference group but also across different stages of optic neuropathy. This finding supports the hypothesis that blood serum serine levels in patients with type 2 diabetes could serve as a prognostic biomarker for the development and progression of optic neuropathy.

## 4. Discussion

Analysis of blood serum from patients with type 2 diabetes following vitreoretinal interventions showed a significant decrease in the concentrations of several amino acids. In particular, we found decreases in the levels of serine, arginine, proline, and cystine (*p* < 0.001). These findings prompted us to further investigate whether changes in amino acid concentrations were associated with clinical parameters. We therefore analyzed the relationships between serum amino acid levels and indicators of visual function and retinal structural changes, including visual field impairment and thinning of the retinal nerve fiber layer. Among the amino acids analyzed, only serum serine levels were significantly associated with the severity of retinal sensitivity loss and the degree of optic neuropathy.

Serine is a nonessential amino acid that can be either internalized by cells or synthesized de novo from glycolytic intermediates via the serine synthesis pathway (SSP). Serine participates in numerous metabolic processes critical for maintaining cellular homeostasis, proliferation, and physiological cell function [11,22]. Previous research [11] demonstrated that maintaining mice on a serine- and glycine-deprived diet for several months leads to loss of retinal function and peripheral sensitivity. These pathological alterations are partially driven by the production of atypical 1-deoxysphingolipids via serine palmitoyltransferase (SPT) [23]; nonetheless, serine deficiency remains the primary root cause of these impairments.

Given that serine is a nonessential amino acid, its systemic levels are sustained not only by de novo synthesis but also by dietary intake, replenishing the plasma pool through distinct metabolic pathways [11]. Furthermore, the authors demonstrated that the systemic circulation is the primary source of serine for the retina, whereas glycine cleavage and subsequent conversion to serine further elevate retinal serine concentrations. Notably, dietary supplementation with high doses of serine alleviated both retinal and peripheral neuropathy in mice, effectively rescuing the phenotype even in animals with genetic defects that inherently induce systemic serine deficiency [11].

In the present study, a profound decline in blood serum serine levels was observed in patients who had undergone vitreoretinal interventions and presented with confirmed optic neuropathy; notably, the severity of optic nerve damage closely mirrored the reduction in serine content. The compensatory mechanism of serine synthesis via glycine cleavage appears insufficient, as evidenced by a statistically significant decrease in glycine content relative to the reference group (Table 2), leaving serine levels critically low. While Lim et al. concluded that disrupted serine homeostasis may manifest as age-related neuroretinal dysfunction [11], it is hypothesized here that decreased serine levels could serve as a dual prognostic biomarker: indicating the early onset of optic neuropathy in type 2 diabetes and predicting further neuropathy progression during later stages, which can be accurately monitored using highly sensitive techniques such as optical coherence tomography (OCT). Consequently, these findings lay the groundwork for future studies that track plasma serine kinetics in patients with type 2 diabetes at the earliest stages of the disease, as well as evaluating the therapeutic impact of dietary serine supplementation in preventing or mitigating optic neuropathy progression.

## 5. Conclusions

The findings suggest that the decline in blood serum serine levels in patients with type 2 diabetes following vitreoretinal interventions may be associated with the severity of optic neuropathy diagnosed via optical coherence tomography (OCT). Although these observations require further validation through larger cohort studies with appropriate control groups, these preliminary results highlight the potential importance of serine levels in the pathogenesis and diagnosis of retinopathy.

## 6. Limitations of the Study

Although statistically significant differences were observed across several amino acids, this study focused exclusively on changes in serine levels as a potential marker of neuropathy. Consequently, the dynamics of other amino acids that exhibited significant variations were not evaluated. Furthermore, the impact of a serine-supplemented diet on the severity of neuropathy remains to be investigated.

A more comprehensive study design would benefit from the inclusion of four appropriately matched control groups, comprising patients with diabetes without optic neuropathy, patients with optic neuropathy without diabetes or prior surgery, and healthy individuals. Such comparisons would help to better distinguish the respective contributions of diabetes, optic neuropathy, and vitreoretinal intervention to the observed changes in serum amino acid profiles.

## Figures and Tables

**Table 1:** Mean perimetric indices and the number of thinned peripapillary retinal nerve fiber layer (RNFL) segments in the patient cohort. MD—Mean Deviation; PSD—Pattern Standard Deviation; VFI—Visual Field Index; OCT—Optical Coherence Tomography.

Parameters	Mean ± SD (*n* = 28)	Normal Value [13,14,15]
MD (dB)	−16.09 ± 8.0	−2 dB–+2 dB
PSD (dB)	7.73 ± 3.0	<2 dB
VFI (%)	60.00 ± 27	100.00
Number of thinned RNFL sectors (via OCT)	5.0 ± 3.0 out of 12.0	0.0 out of 12.0

**Table 2:** Amino acid profiles of blood serum in the reference group (Ref) and patients with type 2 diabetes mellitus after vitreoretinal surgery (D).

Amino Acids	Reference Group (Ref.) (*n* = 12)	Diabetic Group (D) (*n* = 28)	*p*-Value
Serine	7.16	4.47	<0.001 *
Lysine	8.69	8.67	0.836
Histidine	5.32	4.19	0.003 *
Arginine	6.05	3.77	<0.001 *
Ornithine	5.37	6.23	0.132
Aspartic acid	2.39	1.37	0.165
Threonine	5.82	4.59	0.008 *
Glutamic acid	4.68	6.86	0.016 *
Proline	4.70	8.75	<0.001 *
Glycine	8.22	6.68	0.007 *
Alanine	11.86	12.08	0.813
Cystine	2.47	6.57	<0.001 *
Valine	8.98	8.27	0.111
Methionine	1.19	1.61	0.014 *
Isoleucine	2.27	3.49	0.008 *
Leucine	5.44	5.43	0.723
Tyrosine	3.67	3.50	0.360
Phenylalanine	4.35	3.29	0.006 *
Tryptophan	n.d.	n.d.	—
Hydroxyproline	n.d.	n.d.	—

Note. Data are presented as medians (expressed as percentages of the total amino acid pool). n.d., not detected. * Statistically significant difference between groups according to the Mann–Whitney *U* test (*p* < 0.05).

## Data Availability

The data presented in this study are available on request from the corresponding authors. The data are not publicly available to protect patients’ personal information in accordance with the clinic’s rules.

## Abbreviations

OCT	Optical Coherence Tomography
RNFL	Retinal Nerve Fiber Layer
MD	Mean Deviation
PSD	Pattern Standard Deviation
T2DM	Type 2 diabetes mellitus
VFI	Visual Field Index
DN	Diabetic neuropathy
DSPN	Distal symmetrical polyneuropathy
DPN	Diabetic peripheral neuropathy
DAN	Diabetic Autonomic Neuropathy
NEAA	Non-essential amino acid
